# Application of Rough Set Theory to Improve Outpatient Medical Service Quality in Public Hospitals Based on the Patient Perspective

**DOI:** 10.3389/fpubh.2021.739119

**Published:** 2021-11-24

**Authors:** Man-Li Du, Tao-Hsin Tung, Ping Tao, Ching-Wen Chien, Yen-Ching Chuang

**Affiliations:** ^1^Huadu District of Guangzhou Maternal and Child Health Hospital (Huzhong Hospital), Guangzhou, China; ^2^Enze Medical Research Center, Affiliated Taizhou Hospital of Wenzhou Medical College, Taizhou, China; ^3^Department of Medical Affairs and Planning, Section of Medical Fees Kaohsiung Veterans General Hospital, Kaohsiung City, Taiwan; ^4^Institute for Hospital Management, Tsing Hua University, Shenzhen, China; ^5^Institute of Public Health & Emergency Management, Taizhou University, Taizhou, China; ^6^Business College, Taizhou University, Taizhou, China

**Keywords:** service quality, outpatient satisfaction, multiple attribute decision-making (MADM), data mining, rough set theory (RST)

## Abstract

**Purpose:** To analyze the key factors and decision-making behaviors affecting overall satisfaction based on perceptual data of outpatients.

**Methods:** The official satisfaction questionnaire developed by the National Health Commission of the People's Republic of China was used. Rough set theory was used to identify the perception patterns between condition attributes (i.e., service factors) and a decision attribute (i.e., overall service level) and to express them in rule form (i.e., if-then).

**Results:** The four minimal-coverage rules, with strength exceeding 10% in the good class, and six crucial condition attributes were obtained: “Ease of registration (*C*_1_),” “Respected by registered staff (*C*_2_),” “Registered staff's listening (*C*_3_),” “Respected by doctor (*C*_9_),” “Signpost (*C*_12_),” and “Privacy (*C*_16_).” In addition, the average hit rate for 5-fold cross-validation was 90.86%.

**Conclusions:** A series of decision rules could help decision-makers easily understand outpatients' situations and propose more suitable programs for improving hospital service quality because these decision rules are based on actual outpatient experiences.

## Introduction

Hospitals are a service industry whose medical service directly affects patient's lives, medical treatment, and health care (1). Outpatient service is the main medical service provided by hospitals, and most outpatients usually need medium- or long-term diagnosis and treatment. Furthermore, when a hospital meets the needs of existing patients, it attracts more potential patients (2, 3). For these reasons, it is crucial for hospital management to understand and improve the satisfaction of outpatients (4–8).

Hospital service quality involves both qualitative and quantitative factors, which belong to multi-attribute decision-making (MADM). For example, Altuntas et al. developed a MADM method based on a weighted SERVQUAL scale for measuring the perceived service quality of hospitals (5). Shieh et al. developed the modified Decision Making Trial and Evaluation Laboratory (DEMATEL) method to identify the key factors for medical service quality (9). Fei et al. proposed an extended best–worst multi-criteria decision-making method using belief functions and their applications in hospital service evaluation (4). Then, considering the fuzziness of expert's opinion expression, some scholars proposed various fuzzy MADM models. For example, Chen and Hung developed a fuzzy MADM model with interval type-2 fuzzy sets for hospital service quality assessment (10). Tuzkaya et al. developed a hybrid fuzzy MADM model to evaluate hospital service quality based on the interval-valued intuitionistic fuzzy-preference ranking organization method for enrichment evaluations (IVIF-PROMETHEE) (11). Li and He used the 2-tuple MADM method for hospital service quality assessment with linguistic preferences (12). These models are mainly based on expert knowledge as the decision-making basis of service quality evaluation. This decision-making method can easily obtain evaluation and improvement strategies. However, the results are also easily influenced by expert's practical experience and limited knowledge, which does not necessarily conform to the behavior pattern of patient satisfaction.

To fill the gap, this study applied a data-mining method to establish behavior patterns of outpatient patient satisfaction from hospital service quality survey data. First, the questionnaire developed by the National Health Commission of the People's Republic of China was used to investigate the service quality for a third-level first-class hospital. Then, based on the survey data, the decision model between service factors and overall service level was established using rough set theory (RST). Finally, this decision-making model (i.e., data-driven knowledge model) is helpful for hospital quality management departments to propose various improvement directions and measures.

This paper is structured as follows: Section 2 introduces the questionnaire, RST, and data collection. Section 3 describes the case study results based on RST. Section 4 discusses management practices, model stability, and research limitations. Section 5 presents the conclusions.

## Materials and Methods

### The Public Hospital Satisfaction Questionnaire

In China, to improve the quality of medical services in nationwide public hospitals, the National Health Commission set up a national satisfaction survey management platform and designed a series of satisfaction questionnaires. The questionnaire includes outpatient, inpatient, and medical staff versions. Subsequently, a large medical satisfaction survey was conducted in all two- and three-level public hospitals in the country. The outpatient satisfaction questionnaire was used as the purpose of this study is to improve the satisfaction of outpatient medical services from the perspective of outpatient behavior patterns. The questionnaire consists of two parts: service quality factors (i.e., condition attributes defined in RST) and overall service level (i.e., decision attribute defined in rough set). In addition, the overall satisfaction value originates from the average value of all service quality factors (i.e., condition attributes) and is divided equally into three levels: the first third (marked as “Good class [*D* = 1]”), the middle third (marked as “Medium class [*D* = 2]”), and the bottom third (marked as “Poor class [*D* = 3]”). The public hospital satisfaction questionnaire in this study is shown in Table 1.

**Table 1:** The public hospital satisfaction questionnaire.

**Attributes**	**Description**	**Domain values**
***Condition attributes***		
**Registration**		
Ease of registration (*C*_1_)	Patient or family members can easily complete the registration procedure through the outpatient registration system.	{Not satisfied = 1; somewhat dissatisfied = 2; somewhat satisfied = 3; satisfied = 4}
Respected by registered staff (*C*_2_)	Registered staff serve patients and their families with an equal attitude.	{Not satisfied = 1; somewhat dissatisfied = 2; somewhat satisfied = 3; satisfied = 4}
Registered staff's listening (*C*_3_)	Registered staff carefully listen to the patients and their families.	{Not satisfied = 1; somewhat dissatisfied = 2; somewhat satisfied = 3; satisfied = 4}
**Pharmacy**		
Respected by pharmacy staff (*C*_4_)	Pharmacy staff serve patients and their families with an equal attitude	{Not satisfied = 1; somewhat dissatisfied = 2; somewhat satisfied = 3; satisfied = 4}
Pharmacy staff's listening (*C*_5_)	Pharmacy staff carefully listen to the patients and their families.	{Not satisfied = 1; somewhat dissatisfied = 2; somewhat satisfied = 3; satisfied = 4}
**Nurse**		
Respected by nurse (*C*_6_)	Nurse serves patients and their families with an equal attitude.	{Not satisfied = 1; somewhat dissatisfied = 2; somewhat satisfied = 3; satisfied = 4}
Nurse's listening (*C*_7_)	Nurse carefully listens to the patients and their families.	{Not satisfied = 1; somewhat dissatisfied = 2; somewhat satisfied = 3; satisfied = 4}
Nurse's expression (*C*_8_)	Nurse carefully explains to the patients and their families, and they clearly understand the meaning of the expression.	{Not satisfied = 1; somewhat dissatisfied = 2; somewhat satisfied = 3; satisfied = 4}
**Doctor**		
Respected by Doctor (*C*_9_)	Doctor serves patients and their families with an equal attitude.	{Not satisfied = 1; somewhat dissatisfied = 2; somewhat satisfied = 3; satisfied = 4}
Doctor's listening (*C*_10_)	Doctor carefully listens to the speak content of the patients and their families.	{Not satisfied = 1; somewhat dissatisfied = 2; somewhat satisfied = 3; satisfied = 4}
Doctor's speaking (*C*_11_)	Doctor carefully explains to the patients and their families, and they clearly understand the meaning of the expression.	{Not satisfied = 1; somewhat dissatisfied = 2; somewhat satisfied = 3; satisfied = 4}
**Hospital facilities**		
Signpost (*C*_12_)	Signs and directions in the hospital are clear	{Not satisfied = 1; somewhat dissatisfied = 2; somewhat satisfied = 3; satisfied = 4}
Hardware (*C*_13_)	General impression of hospital hardware facilities (including seats, elevators, drinking water equipment, etc.)	{Not satisfied = 1; somewhat dissatisfied = 2; somewhat satisfied = 3; satisfied = 4}
Toilet clean (*C*_14_)	The toilet is clean and free of odor.	{Not satisfied = 1; somewhat dissatisfied = 2; somewhat satisfied = 3; satisfied = 4}
Space allocation (*C*_15_)	Space allocation and route planning make people feel comfortable and convenient.	{Not satisfied = 1; somewhat dissatisfied = 2; somewhat satisfied = 3; satisfied = 4}
**Diagnosis**		
Privacy (*C*_16_)	Medical staff always pay attention to protecting personal privacy, during diagnosis and treatment.	{Not satisfied = 1; somewhat dissatisfied = 2; somewhat satisfied = 3; satisfied = 4}
Timely response (*C*_17_)	Medical staff always pay attention to complaints and resolve problems, during diagnosis and treatment.	{Not satisfied = 1; somewhat dissatisfied = 2; somewhat satisfied = 3; satisfied = 4}
***Decision attribute***		
Overall satisfaction	Outpatients' overall satisfaction with the services provided.	{Good = 1; medium = 2; poor = 3}

### A Brief Introduction to RST

Pawlak discovered the imprecise fuzzy relations between condition attributes and a decision attribute in classification problems and then developed a data mining method, RST (13). The basic concept of this method is to approximate the goal based on the equivalence relationship between attributes and to show the behavior/decision pattern behind the data in the form of rules (14). Because of this concept, the method has a unique advantage that it can directly evaluate and analyze quantitative and qualitative attributes without knowing the probability distribution of the data (i.e., the probability distribution in statistics) before data analysis (15). Accordingly, this method plays a key role in the fields of artificial intelligence and cognitive science and is applied to many different topics, such as finance investment (16), consumer behavior (17), and human resource development (18). The basic definition and brief calculation of RST are shown in Supplementary Appendix A (17, 19).

### Participants and Data Collection

**Figure 1:**
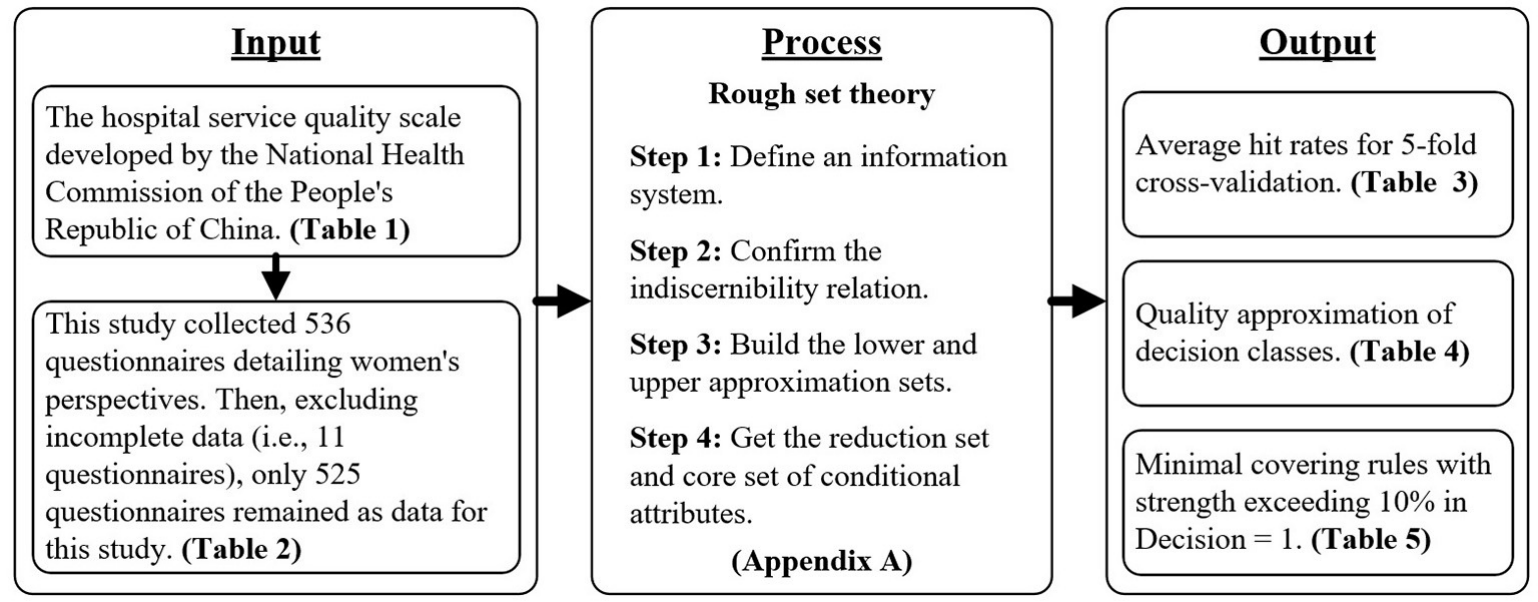
The research flow chart.

This study was conducted following the principles of the institutional ethics committee and in accordance with the Declaration of Helsinki. All participant's information was kept anonymous. This observational study was approved by the Guangdong Nursing Association on November 1, 2018 (No. hdfyhlbgdhlxueh2019zx113). The questionnaire survey was conducted in March 2019. This study collected 536 questionnaires detailing women's perspectives. After excluding questionnaires with incomplete data (i.e., 11 questionnaires), 525 questionnaires remained as data for this study. Finally, this study uses the rose2 software to do the rough set analysis. The detailed information regarding the background of respondents is shown in Table 2. The research flowchart is shown in Figure 1.

**Table 2:** Respondent background information for the case study.

	**Sample size**	**Frequency (%)**
**Gender**		
Male	0	0%
Female	525	100%
**Age**		
Under 30	246	47%
30–39	250	48%
40 and above	29	5%
**Education**		
High school and below	242	46%
College and above	283	54%
**Payment**		
At one's own expense	172	33%
New rural cooperative medical	52	10%
Basic medical insurance system (urban employee/ residents)	261	50%
Publicly funded free medical care	40	7%

### The Robustness of RST Model

To further determine the reliability of the decision rules generated in this study, a 5-fold cross-validation method was applied to the dataset. First, 80% of the dataset was randomly selected as training samples to generate decision rules. Next, the remaining 20% of the data was used as a test sample to verify the click rate of the generated decision rules (i.e., the percentage of the correct predictions for each decision class). Finally, these processes were repeated five times through RST modeling; their average hit rate is shown in Table 3. In addition, this method was compared with four well-known data mining methods: decision tree, random forest (RF), back-propagation artificial neural network (BPANN), and the support vector machine (SVM).

**Table 3:** Average hit rates for 5-fold cross-validation.

	**RST/DT/RF/BPANN/SVM**		
**No**.	**Good class (*D =* 1)**	**Medium class (*D =* 2)**	**Poor class (*D =* 3)**
Good class (*D =* 1)	173/171/169/165/158	2/28/9/2/8	0/2/0/0/0
Medium class (*D =* 2)	19/23/5/4/9	141/116/166/175/172	15/15/22/9/9
Poor class (*D =* 3)	0/0/0/0/0	12/21/5/9/8	163/149/149/161/161
Correctly classified (%)	90.86/83.05/92.19/95.43/93.52		

## Results

### Quality of Approximation Boundary

The overall quality of the decision class approximation boundary is 0.9733, and the approximation accuracy for each class is shown in Table 4. The results showed that the classification boundary of the global decision has a high approximation quality. First, for “Good class (*D* = 1),” the lower and upper approximations are 175 objects each for an approximation quality of 1.000 (175/175). Then, for “Medium class (*D* = 2),” the lower approximation is 166 objects, and the upper approximation is 180 objects for an approximation quality of 0.9222 (166/180). Last, for “Poor class (*D* = 3),” the lower approximation is 170 objects, and the upper approximation is 184 objects for an approximation quality of 0.9239 (170/184). From the approximation accuracy perspective, the approximate boundary of class 1 is clear and has no ambiguity (i.e., no roughness). In addition, the approximate boundaries of classes 2 and 3 are fuzzy—that is, there is an ambiguous phenomenon (i.e., roughness).

**Table 4:** Quality approximation of decision classes.

**Class**	**No. of objects**	**Lower approximation**	**Upper approximation**	**Accuracy**
Good class (D=1)	175	175	175	1
Medium class (D = 2)	175	166	180	0.9222
Poor class (*D =* 3)	175	170	184	0.9239

### Decision Rules

RST can produce minimal-coverage rules, 52 rules in this dataset. Among these, 8 rules apply to “Good class (*D* = 1),” 28 apply to “Medium class (*D* = 2),” and 16 apply to “Poor class (*D* = 3).” To better achieve the goal of improving outpatient's satisfaction with hospital service quality, this study focused only on “Good class (*D* = 1),” and the data percentage threshold for this class was set at 10%. The four rules included indicate that, if the requirements of conditional attributes are met, the hospital's overall service satisfaction has a certain probability of reaching “Good class (*D* = 1).” The rules also represent four ways to improve the quality of hospital services and thus achieve the service levels expected by outpatients. The four rules were selected as shown in Table 5.

**Table 5:** Minimal covering rules with strength exceeding 10% in Decision = 1.

**No**.	**Conditions**	**Decision**	**Number of objects**
1	(*C*_3_ = 4) ”3026(*C*_12_ = 4) ”3026(*C*_13_ = 4) ”3026(*C*_17_ = 4)	Good	128/225 (73.14%)
2	(*C*_1_ = 4) ”3026(*C*_3_ = 4) ”3026(*C*_6_ = 4) ”3026(*C*_9_ = 4) ”3026(*C*_11_ = 4) ”3026(*C*_16_ = 4)	Good	119/225 (68.00%)
3	(*C*_1_ = 4) ”3026(*C*_2_ = 4) ”3026(*C*_9_ = 4) ”3026(*C*_15_ = 4) ”3026(*C*_16_ = 4)	Good	102/225 (58.29%)
4	(*C*_1_ = 4) ”3026(*C*_2_ = 4) ”3026(*C*_5_ = 4) ”3026(*C*_10_ = 4) ”3026(*C*_12_ = 4) ”3026(*C*_14_ = 3)	Good	38/225 (21.71%)

*The values in the parentheses of the number of objects refer to the relative strength*.

Rule 1 indicated that 73.14% of hospital outpatients rated the overall service evaluation of the hospital as “Good class (*D* = 1),” which has the condition attributes of “Registered staff's listening (*C*_3_) = {4},” “Signpost (*C*_12_) = {4},” “Hardware (*C*_13_) = {4},” and “Timely response (*C*_17_) = {4}.” Rule 2 indicated that 68.00% of hospital outpatients associated the “Good class (*D* = 1)” service rating with “Ease of registration (*C*_1_) = {4},” “Registered staff's listening (*C*_3_) = {4},” “Respected by nurse (*C*_6_) = {4},” “Respected by doctors (*C*_9_) = {4},” “Doctor's speaking (*C*_11_) = {4},” and “Privacy (*C*_16_) = {4}.” Rule 3 indicated that 58.29% of hospital outpatients associated the “Good class (*D* = 1)” service rating with “Ease of registration (*C*_1_) = {4},” “Respected by registered staff (*C*_2_) = {4},” “Respected by doctors (*C*_9_) = {4},” “Space allocation (*C*_15_) = {4},” and “Privacy (*C*_16_) = {4}.” Finally, Rule 4 indicated that 21.71% of hospital outpatients associated the “Good class (*D* = 1)” service rating with “Ease of registration (*C*_1_) = {4},” “Respected by registered staff (*C*_2_) = {4},” “Pharmacy staff's listening (*C*_5_) = {4},” “Doctor's listening (*C*_10_) = {4},” “Signpost (*C*_12_) = {4},” and “Toilet clean (*C*_14_) = {4}.”

To further determine the relative importance of the condition attributes related to “Good (*D* = 1),” the frequency of these condition attributes was as follows: “Ease of registration (*C*_1_),” three times; “Respected by registered staff (*C*_2_),” “Registered staff's listening (*C*_3_),” “Respected by doctor (*C*_9_),” “Signpost (*C*_12_),” and “Privacy (*C*_16_),” two times; “Pharmacy staff's listening (*C*_5_),” “Respected by nurse (*C*_6_),” “Doctor's listening (*C*_10_),” “Doctor's speaking (*C*_11_),” “Hardware (*C*_13_),” “Toilet clean (*C*_14_),” “Space allocation (*C*_15_),” and “Timely response (*C*_17_),” one time. These results indicate that “Ease of registration (*C*_1_),” “Respected by registered staff (*C*_2_),” “Registered staff's listening (*C*_3_),” “Respected by doctor (*C*_9_),” “Signpost (*C*_12_),” and “Privacy (*C*_16_)” are several major condition attributes that outpatients recognize in hospitals with good medical quality.

### A Correct Classification Rate of age Groups

In order to further understand the classification accuracy of age groups-based data. The 525 data set is divided into two groups according to age: (i) under 30 years old (Sample size: 246, e.g., 47%); and (ii) over 30 years old (Sample size: 279, e.g., 53%). Based on the results of 5-fold cross-validation, the classification accuracy of the former is 83.32%; The latter is 86.4%. Compared with the original classification accuracy (i.e., 90.86%, all sample sizes: 525), the data according to different age groups will really affect the accuracy of classification. Since the interviewees are all women, there are no men. Therefore, this study did not make a comparative analysis of gender on the results. The results are shown in Table 6.

**Table 6:** The average hit rate of 5-fold cross-validation in different age groups.

**Age groups**	**Under 30 years / Over 30 years**		
**No**.	**Good class (*D =* 1)**	**Medium class (*D =* 2)**	**Poor class (*D =* 3)**
Good class (*D =* 1)	74/93	3/5	0/0
Medium class (*D =* 2)	16/16	64/57	11/11
Poor class (*D =* 3)	0/0	11/6	67/91
Correctly classified (%)	83.32/86.4		

## Discussion

### Management Practice

This study indicates the six most important attributes clinical practice: ease of registration, respected by registered staff, registered staff's listening, respected by doctor, signpost, and privacy. This indicates that patients would like to complete the outpatient process smoothly. First, the online reservation system should be easy to use, and telephone reservation personnel need to be empathetic and listen carefully to the patient's needs so that they can successfully complete the reservation process. This is because, once patients miss their appointment opportunity, they must spend more time waiting for an appointment. In addition, physical condition is a very private issue. Patients hope that clinicians can respect their wishes or opinions in the diagnosis or treatment process and protect their privacy. Finally, the hospital should have a clear movement route with signposts to help patients find the visiting area quickly and save time, especially for pregnant women for whom moving is not convenient. As mentioned above, these six service quality factors are critical and foundational to overall satisfaction with hospitals. In addition, hospital decision-makers can choose rules 1 to 4 to improve the overall service quality of outpatient service according to the actual situation.

The results indicate that the difference in modeling quality between the five methods is not significantly based on the same data. However, the RST used in this study can demonstrate the pattern between condition attributes and a decision attribute through the regular expression. This way of expressing rules can assist hospital decision-makers or managers to more easily understand the decision rules for each decision class and their probability of occurrence. Compared with RST, the BPANN method requires more training time (16, 20, 21). Furthermore, these RF, BPANN, and SVM methods cannot provide this information because they belong to the black-box algorithms category (22).

### Research Limitations

The limitations of this study were as follows. (i) The questionnaire items were used by the National Health Commission of the People's Republic of China, without considering other questionnaire scales. (ii) The questionnaire survey was conducted in March 2019, and the analysis of the results was limited to the satisfaction analysis from the female perspective, without considering other factors such as related events, gender, grade, and admission time. The reason is that other factors may affect the change of classification accuracy so they are not within the scope of this study. (iii) The results of RST analysis are limited to the behaviors of the investigated participants and should not be extended to other hospitals.

## Conclusions

This is the first time to study the public hospital service quality scale based on the National Health Commission of the People's Republic of China, and apply rough set theory to explore the key attributes and behavior rules that affect the overall service quality from a group of patient behavior data. The key attributes can help decision makers better understand the factors that patients care most about; Behavior rules can help decision-makers put forward specific improvement strategies. As a beginning, this study provides different thinking of data behavior decision-making from expert experience. Chinese public hospitals can take this research model as the research basis, and consider other factors such as related events, gender, grade, and admission time, so as to provide more in-depth results and analysis in the future.

## Data Availability Statement

The original contributions presented in the study are included in the article/Supplementary Material, further inquiries can be directed to the corresponding authors.

## Ethics Statement

The studies involving human participants were reviewed and approved by Guangdong Nursing Association (No. hdfyhlbgdhlxueh2019zx113). Written informed consent for participation was not required for this study in accordance with the national legislation and the institutional requirements.

## Author Contributions

M-LD and PT collected the data and wrote a draft article. Y-CC calculated the RST results, interpreted the results, and revised the article. T-HT and C-WC designed the research process and content and reviewed the final version. All authors have read and agreed to the published version of the manuscript.

## Funding

This research was funded by a study on nurse-patient relationship and patient satisfactions in a women and children's hospital, Grant No. gdhlxueh2019zx113.

## Conflict of Interest

The authors declare that the research was conducted in the absence of any commercial or financial relationships that could be construed as a potential conflict of interest.

## Publisher's Note

All claims expressed in this article are solely those of the authors and do not necessarily represent those of their affiliated organizations, or those of the publisher, the editors and the reviewers. Any product that may be evaluated in this article, or claim that may be made by its manufacturer, is not guaranteed or endorsed by the publisher.
