# Supplementary material for: Application of Rough Set Theory to Improve Outpatient Medical Service Quality in Public Hospitals Based on the Patient Perspective
Source: Front Public Health. 2021 Nov 24;9:739119. doi: 10.3389/fpubh.2021.739119 (PMC8654147; doi:10.3389/fpubh.2021.739119)
Supplement: Supplementary file 1 [file Data_Sheet_1.docx]

Supplementary Material

# Appendix A: Rough set theory

## Step 1: Define an information system

The information system $S$ includes four elements, as shown in Equation (1):

$S=\left(U,A,V,f\right)$ (1)

where:

$U$ is a nonempty finite set (also called the universal set) with $n$ objects $\left\{u_{1},u_{2},\ldots,u_{n}\right\}$;

$A$ is a nonempty finite set with $m$ properties/attributes, which can be further disassembled into the condition attribute $C$ and decision attribute $D$; $A$ can also be defined as $A=C\cup D=\left\{c_{1},c_{2},\ldots,c_{m},d\right\}$;

$V$ is the domain value set of $A$ ; and

$f$ is the information description function (i.e., $f:U\times A\to V$), such as $f(p,g)\in V$, in which $p\in U,\text{ }g\in A$.

## Step 2: Confirm the indiscernibility relation

The nonempty finite set $U$ through conditions and decision attributes can be divided into two subsets: $U/C$ and $U/D$. $z$ and $y$ are two objects treated as a subset of attributes $R\subseteq A$ , in which the indiscernibility relation of the attribute set $A$ is defined as shown in Equation (2):

$IND(R)=\left\{\left(z,y\right)\in U\times U,\forall g\in R,\text{ }f\left(z,g\right)=f\left(y,g\right)\right\}$ (2)

where $IND(R)$ can be used to divide $U$ into several partitions (i.e., $U/IND(R)=\left\{I_{R}\left|z\in U\right.\right\}$, in which $I_{R}$ is the equivalence class of an object that comprises all objects $y\in U$ such that $z$ is indiscernible with $y$ based on the condition attribute subset $R$).

## Step 3: Build the lower and upper approximation sets

Let $R\subseteq A$ and $z\in U$. The lower and upper approximations of $Z$ in the information system and other related definitions are shown in Equations (3)–(6):

The lower approximation: $\underline{R}Z=\{z\in U|I_{R}[z]\subseteq Z\}$ (3)

The upper approximation: $\overline{R}Z=\{z\in U|I_{R}[z]\cap Z\neq\emptyset\}$ (4)

The boundary: $BN_{R}(Z)=\overline{R}Z-\underline{R}Z$ (5)

The quality of approximation: $\alpha_{R}(Z)=\frac{card(\underline{R}Z)}{card(\overline{R}Z)}$ (6)

where $card(\text{ })$ is the cardinality of a set.

Let $D=\left\{Z_{1},Z_{2},\ldots,Z_{n}\right\}$ be a classification of $U$ (i.e., $Z_{i}$ are called classes of $D$). The lower and upper approximation of $D$ is by $R\subseteq A$. The related definitions are shown in Equations (7)–(9):

The lower approximation: $\underline{R}D=\{\underline{R}D_{1},\underline{R}D_{2},\ldots\text{,}\underline{R}D_{n}\}$ (7)

The upper approximation: $\overline{R}D=\{\overline{R}D_{1},\overline{R}D_{2},\ldots\text{,}\overline{R}D_{n}\}$ (8)

The quality of approximation: $\beta_{R}(D)=\frac{\sum_{i=1}^{n} card(\underline{R}Z_{i})}{card(U)}$ (9)

where $\beta_{R}(D)$ is also the ratio of all $D$-correctly classified objects to all objects in the system.

## Step 4: Get the reduction set and core set of conditional attributes

In the information table (i.e., an information system), certain attributes may be redundant. Therefore, these attributes can be deleted without losing the necessary classification information. The classification quality provided by the subset $R\subseteq A$ is the same as that provided by the original attribute set $A$—that is $\beta_{R}(F)=\beta_{A}(F)$, which is also called the $F$-reduction of $R$, is represented by $RED_{F}(R)$. The reduction in conditional attributes is a minimal subset, which allows the universal elements to maintain the same classification level with the entire attribute set. In other words, in terms of the classification of universe elements, these nonreduced condition attributes are redundant. Besides, the core is the common attribute of all reduction sets in the condition attribute, which means that it is the most essential or fundamental attribute set in the decision table.

## Step 5: Derive the decision rules and their corresponding strengths

The reduction of the condition attribute set highlights the relevant relationship between condition attributes and decision classes. As such, the decision rule can be derived from the decision table for decision analysis. This rule is also called the rule of minimum coverage. The expression of the decision rules is shown in Equation (10):

A decision rule in the decision table is $\Theta\to\Gamma$, which is also read as $\Theta$ then $\Gamma$ (10)

where $\Theta$ and $\Gamma$ represent the condition attributes and a decision attribute of the rules, respectively. The decision rules are “if-then” statements related to conditions and decision categories. It also expresses the relationship between condition attributes and decision attributes. The strength of decision rules is shown in Equation (11):

$\eta_{s}\left(\Theta,\Gamma\right)=\frac{\sup_{S}\left(\Theta,\Gamma\right)}{card\left(U\right)}$ (11)

where:

$\sup_{S}\left(\Theta,\Gamma\right)$ supports the decision rule $\Theta\to\Gamma$ in $S$, and

$card\left(U\right)$ is the cardinality of $U$.

The strength of decision rules can be simply expressed as a ratio, which represents the number of decision rules divided by the total numbers in the data table. It is essential for decision-makers to understand how often each rule appears in social behavior. Moreover, this value comes from real responses, not the hypothesis.
